# A Care Knowledge Management System Based on an Ontological Model of Caring for People With Dementia: Knowledge Representation and Development Study

**DOI:** 10.2196/25968

**Published:** 2021-06-08

**Authors:** Gyungha Kim, Hwawoo Jeon, Sung Kee Park, Yong Suk Choi, Yoonseob Lim

**Affiliations:** 1 The Research Center for Diagnosis, Treatment and Care System of Dementia Korea Institute of Science and Technology Seoul Republic of Korea; 2 Carbon & Light Materials Application Group Korea Institute of Industrial Technology Jeonju Republic of Korea; 3 Department of Computer Science Hanyang University Seoul Republic of Korea; 4 Department of HY-KIST Bio-convergence Hanyang University Seoul Republic of Korea

**Keywords:** caregiver, caregiver for person with dementia, knowledge model, ontology, knowledge management, semantic reasoning

## Abstract

**Background:**

Caregivers of people with dementia find it extremely difficult to choose the best care method because of complex environments and the variable symptoms of dementia. To alleviate this care burden, interventions have been proposed that use computer- or web-based applications. For example, an automatic diagnosis of the condition can improve the well-being of both the person with dementia and the caregiver. Other interventions support the individual with dementia in living independently.

**Objective:**

The aim of this study was to develop an ontology-based care knowledge management system for people with dementia that will provide caregivers with a care guide suited to the environment and to the individual patient’s symptoms. This should also enable knowledge sharing among caregivers.

**Methods:**

To build the care knowledge model, we reviewed existing ontologies that contain concepts and knowledge descriptions relating to the care of those with dementia, and we considered dementia care manuals. The basic concepts of the care ontology were confirmed by experts in Korea. To infer the different care methods required for the individual dementia patient, the reasoning rules as defined in Semantic Web Rule Languages and Prolog were utilized. The accuracy of the care knowledge in the ontological model and the usability of the proposed system were evaluated by using the Pellet reasoner and OntOlogy Pitfall Scanner!, and a survey and interviews were conducted with caregivers working in care centers in Korea.

**Results:**

The care knowledge model contains six top-level concepts: care knowledge, task, assessment, person, environment, and medical knowledge. Based on this ontological model of dementia care, caregivers at a dementia care facility in Korea were able to access the care knowledge easily through a graphical user interface. The evaluation by the care experts showed that the system contained accurate care knowledge and a level of assessment comparable to normal assessment tools.

**Conclusions:**

In this study, we developed a care knowledge system that can provide caregivers with care guides suited to individuals with dementia. We anticipate that the system could reduce the workload of caregivers.

## Introduction

People with dementia exhibit memory loss, personality changes, depression, and instability in daily life, and they become highly dependent on family members and caregivers [1]. If a person has severe memory loss, family members must regularly check whether they are taking their medication, and they need to remind them of the detailed procedures of daily activities such as using the toilet or taking a shower. This high level of dependency demands constant attention and frequently requires additional support from care professionals. For this reason, most of the care costs relate to the need for family and social support rather than to the medical diagnosis [1]. Furthermore, the symptoms of dementia will differ depending on factors such as the living environment and personality [2]. The complexities of the care require a careful assessment of the physical and psychological abilities of the person with dementia and an assessment of the competence of those providing the care. One of the most difficult tasks for dementia caregivers is to choose the most effective approach for meeting the care needs and matching the behavioral characteristics of the individual [1,3-5].

To alleviate the care burden on caregivers, several studies have developed computer-based support systems that improve the well-being of the person with dementia [6-13]. These studies propose systems that support the caregivers by monitoring the health and daily activities of the person with dementia. The ambient-assisted intervention system (AAIS) offers ambient intelligence to improve quality of life by identifying the presence of behavioral and psychological symptoms of dementia (BPSDs) and by suggesting an appropriate intervention for the various symptoms [8]. For example, the AAIS will give repeated reminders to a person who frequently forgets to take their medicine. The K4Care project offers a computer-supported structure that represents the health care procedures needed to assist a person suffering from a particular disease, syndrome, or social issue [10]. In order to improve the quality of treatment that a person with dementia receives, decision support tools that help physicians detect wrong diagnoses, unobserved comorbidities, incomplete descriptions of the patient’s condition, and appropriate prevention measures have been proposed [12,13]. For example, the Dementia Management and Support System (DMSS-R) supports the clinical routines and decision processes implemented by health professionals in their daily practice [12]. However, existing studies mainly focus on the clinical and statistical significance of the psychosocial and environmental interventions used to improve the welfare of persons with dementia [8-13].

Other studies have proposed interventions that can improve the independence of the person with dementia and so provide relief to caregivers [14-16]. A tablet-based app called MapHabit provides a customizable visual map that helps those with various Alzheimer-related disorders. For example, persons with memory loss can obtain step-by-step support in completing their daily activities by themselves while keeping connected with family members [14]. Another study introduced a digital memory notebook (DMN) application that is designed to help individuals with mild cognitive impairment to improve their everyday functioning [15]. The DMN is equipped with daily notes, schedule management, and to-do lists, and it helps patients to plan longer-term goals and to complete their everyday activities. Another study reviews the mobile apps that are designed to support the caregivers of older adults and that can be found on Google Play and iTunes [16]. These mobile apps generally address specific aspects such as information and resources, family communication, memory aids, care for the caregiver, behavioral solutions, medication management safety, or personal health record tracking [16].

On the whole, previous studies have attempted to enhance the physical and mental ability of the individual with dementia by proposing an intelligent system that either diagnoses their medical condition or increases their level of independence in daily activities. It is hoped that as the person with dementia becomes increasingly independent, less attention will be required from caregivers, and that this will eventually result in a reduced care burden for both family members and caregivers. However, since the BPSDs are highly complex and fluctuate sporadically, caregivers will still need to constantly monitor the health condition of the person with dementia and detect any changes in their behavioral symptoms [2,3]. This means that caregivers may have difficulty in choosing an appropriate care method regardless of the symptoms diagnosed previously. It has been suggested that to lessen the care burden on family members and caregivers, new approaches that are tailored to the patients, to the contexts where the symptoms occur, and to the caregivers are required [2,3,7].

In this study, we propose a care knowledge management system that can provide a care guide that is suited to the variable behavioral and psychological symptoms of the person with dementia by using an ontological knowledge model of dementia. The ontology-based model is a computer-interpretable knowledge model for formalizing and representing shared concepts in a specific domain of interest. This is expressed in a language that enables knowledge sharing among different applications and for various reasons [17-19]. The model can help improve data-driven decisions by explicitly defining and providing semantic concepts in a specific domain. Because of these advantages, the knowledge model for the proposed system could represent various care situations, which would include the symptoms of dementia. The Pellet and Prolog reasoner that will be built into the system can infer the proper care approach required for different individuals with dementia [20]. The graphical user interface (GUI) of the system will provide an easy way for family members or caregivers in care centers to share and update care data so that each caregiver can receive appropriate care guidelines simply by sending queries to the system without needing to ask the other caregivers or family members. To evaluate the accuracy of the care knowledge in the ontological model and the usability of the proposed system, we conducted a brief survey and interviews with caregivers working in care centers in Korea.

## Methods

### Determining the Domain and Scope of the Ontological Model of Dementia Care

To determine the domain and scope for the ontological knowledge model of dementia care, we tried to answer the following questions: (1) What is the target and scope to be covered by the ontology? (2) What will the ontology be used for? (3) How should the data be classified within the whole area of data available to users of the knowledge model? (4) How can the model represent knowledge to the target? (5) Who will be the users of the ontology? Based on the responses to these questions, we set the target for our knowledge model as a caregiver who actively cares for a person with mild dementia, who may be a family member, and we set the scope of the knowledge to include environmental information, schedule information, daily activity information, and disease information such as would be necessary to determine a care method for the individual with dementia.

### Consideration of the Reuse of Existing Ontologies

We reviewed existing ontologies that contain various concepts and knowledge descriptions relevant to the care of persons with dementia. From the relevant ontologies, disease information relating to dementia was based on the AAIS [8] and K4Care approaches [10], and concepts involving environmental information were drawn from the KnowRob ontology [11].

### Collecting Terms and Developing the Ontological Model of Dementia Care

We used two different methods to collect the terms and to define the classes for the ontological model: (1) a text-based document about caring for those with dementia and (2) interviews with care experts in the field of dementia. First, we collected the handbooks on dementia care that are already used in dementia care facilities in Korea [21-23]. From these care handbooks, we retrieved several sentences that describe the daily activities of people with dementia and related care methods. These care manuals also contain questions devised to evaluate the physical and psychological condition of the person with dementia and to assess their daily activities. From the sentences and questions collected, we extracted concepts relating to dementia care and then defined a top-level class with a hierarchy of concepts following a top-down approach [18]. A top-down development process starts with a definition of the most general concepts in the domain and is followed by subsequent specializations of the concepts. For example, we started with creating classes for the general concepts of “person,” “assessment,” “task,” “medical knowledge,” “environment,” and “care knowledge.” Then we specialized those classes further by creating their subclasses. The subclasses extracted were then arranged by mapping or by adding them to the class hierarchy. Overall, it took 3 months to build our initial version of the ontological model.

We then updated the terms and classes of the ontological model by interviewing 2 experts at a dementia center in Korea. Both experts had more than 10 years of experience in the dementia center, one as a director and the other as a manager. It took a total of 6 months to update the initial model, including 3 interviews with these experts. For example, we added relationships between the causes of the BPSDs in the “Medical Knowledge” class and the care methods in the “Care Knowledge” class and updated the decision rules based on the information provided during the interviews.

The Web Ontology Language (OWL) 2 language proposed by the World Wide Web Consortium was chosen to describe the ontology model [24]. The naming convention for class labels uses nouns and verbs with the first letter of a word being capitalized and multi-word labels (eg, Take_Meals and Take_Drug) having an underscore inserted between the words. Likewise, multi-word instance labels have an underscore inserted between the words and the instance (eg, Take_Meals_01 and Hypertension_Drug_01). The property names are set in the same way. The ontology was developed using the Protégé-OWL ontology editor [25]. Its consistency was checked using the Pellet reasoner [20] and the OntOlogy Pitfall Scanner (OOPS!) [26].

The two co-first authors were actively engaged in the entire process of building the ontological model. Both of them had previous experience in building an ontological model, and one of them has a PhD degree in ontology.

### Decision Rules 

**Figure 1:**
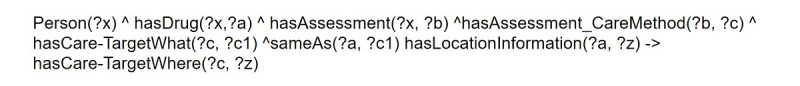
Example of Semantic Web Rule Language rule about location notification.

**Figure 2:**
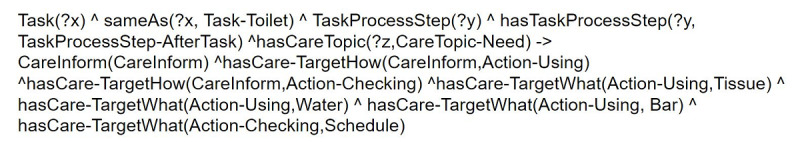
Example of Semantic Web Rule Language rule about Task-Toilet care.

We constructed reasoning rules using the OWL following Protégé, and the semantic rules were created using Semantic Web Rule Language (SWRL) and Prolog [24]. Reasoning rules in SWRL are easily defined within the Protégé-OWL ontology editor, but negative rules cannot be defined. We therefore decided to use SWRL for inferring the general care methods and Prolog for inferring individual care methods that reflect the behavior or psychological condition of a person with dementia. We followed actual care guidelines that we obtained either from interviews with care experts or from care manuals used in the dementia care center in Korea. Based on the information acquired, we designed the decision rules for caring for a person with dementia in different situations and when exhibiting different symptoms of dementia. For example, when retrieving the location of an object, the system utilizes reasoning rules that are defined in SWRL. However, for individual care methods, the system first infers general care methods using SWRL, and then the appropriate care methods for the individual are determined based on the reasoning rules in Prolog. Examples of SWRL rules are presented in Figures 1 and 2. The rules in Figure 1 are used to deduce the location of the hypertension drug for the patient. Person(?x) indicates that the variable x is a person. The binary relation hasDrug (?x, ?a) indicates that person x takes the drug a. If the person takes drug a and the location of the drug is known, the SWRL rule will provide the location of the drug for the person.

The rules in Figure 2 illustrate the process by which care guides direct the toilet use methods and procedures of the person with dementia. By following the rules, the system provides the inference result and informs the individual about the use of tissue, water, toilet rails, and schedule confirmation. The reasoning results from SWRL are refined by the Prolog reasoner, which utilizes the assessment information of the individual. For example, if the person with memory impairment frequently forgets to flush the toilet bowl, the Prolog reasoner will generate an additional care method that reminds the person with dementia to flush the water at the end of using the toilet.

### Evaluation of the Knowledge Management System

We first checked the care ontology with the OOPS! tool [26] to check its compliance with ontology authoring principles in addition to its structural quality. The knowledge model was also evaluated by care experts. Initially, we recruited 30 participants who each had more than a year of experience in caring for a person with dementia. However, only 4 of these participants had professional knowledge of dementia patient care, and we excluded the other 26 participants who had not been trained with professional nursing skills. The 4 participants selected had worked in the same dementia welfare center in Korea for an average of 10 years each, which included periods spent with other organizations. The average age of the participants was 60 years, with a range of 57 to 63 years. The respondents were all female (4/4, 100%). For evaluation, we provided the experts with a video introducing the knowledge management system we had built, followed by an opportunity to use the actual system. After watching the video, the experts were instructed to input patient information, and they then had an opportunity to check or to update the instances generated, such as the location of objects and the assessment data for the patient. A survey and an interview were conducted for 20 minutes after the actual experience with the knowledge management system. The whole evaluation took 30 minutes to an hour. Prior to the test, all participants gave their consent for the use of the experimental results, and the experiment was conducted with privacy protection (IRB number: K-2019-033). During the survey and interview, we asked the experts the following questions: (1) Is the ontology for the care of people with dementia consistent with the knowledge that the care expert holds? (2) Is the ontology for the care of people with dementia sufficient to determine their care guidelines? (3) Are the terms expressed in the ontology the same as the terms as used in actual care knowledge? (4) Is the knowledge management application convenient to use? (5) Does it seem useful for use in a real environment? The responses covered the accuracy, completeness, consistency, and usability of care knowledge on a 5-point scale (1=very invalid, 2=invalid, 3=moderate, 4=valid, and 5=very valid) [27-30]. The content validity index of the answers was calculated by taking the average.

## Results

### Care Ontology for People With Dementia

**Figure 3:**
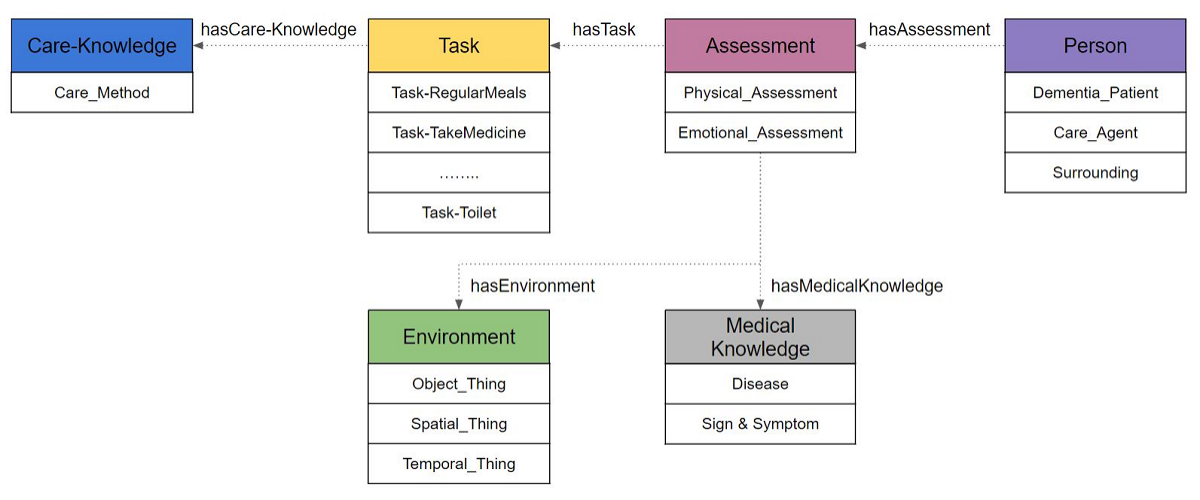
Upper classes of the proposed care ontology for persons with dementia.

The ontological model for caring for people with dementia was based on six different top-level concepts achieved by classifying and defining information that is important in determining the care method needed for a person with dementia: person, task, environment, assessment, medical knowledge, and care knowledge. Figure 3 shows the overall structure of the top-level concepts and the relationships between them. The domain and range of each concept was defined, and the concepts were given properties. The proposed knowledge model has 502 classes and 230 properties.

#### Person Class

The Person class represents the basic profile information on the person with dementia and their caregivers and family members. The basic profile information includes age, address, gender, and social relationship. The Person class was based on a previously developed ontology for supporting chronically ill patients and on the care handbook used in domestic dementia facilities in Korea [10].

#### Task Class

In order to provide a care method for the person with dementia, the activities of their daily life are defined as a Task class, and the Task class has a total of 20 different subtasks. These subtasks cover activities such as eating meals, sleeping, using the toilet, washing, taking medicine, and exercising. To describe the temporal and statistical aspects of each task, the Task class has the properties of task frequency, task execution cycle, task preference, task result, and task step.

#### Environment Class

The Environment class contains three different subclasses: Object_Thing, Spatial_Thing, and Temporal_Thing. Subclass Object_Thing consists of various objects in the living environment of the person with dementia. Subclass Spatial_Thing describes the location of an object, while temporal concepts such as time, birthday, and age are represented in the subclass Temporal_Thing. The structure of this class is based on the existing knowledge model KnowRob, which contains knowledge of various objects in the human environment [11].

#### Assessment Class

The Assessment class covers the behavioral competence of the person with dementia, including their physical ability to perform daily activities at home or in the care facilities and the psychological symptoms of their dementia. Most of the concepts in this class were adopted from the care manual used in welfare centers in Korea and from similar care handbooks [21-23]. The class is organized into the physical and psychological knowledge of the individual, and each instance of the class is associated with a care method described in the Care Knowledge class.

#### Medical Knowledge Class

The Medical Knowledge class is a class that expresses the diseases and symptoms of dementia patients. The basic concepts and structure of this class were adopted from the K4Care ontology and the AAIS ontology [8,10].

#### Care Knowledge Class

The Care Knowledge class describes the various circumstances involved when helping the person with dementia, and most of the concepts defined in this class are linked with the Assessment, Environment, and Medical Knowledge classes.

**Figure 4:**
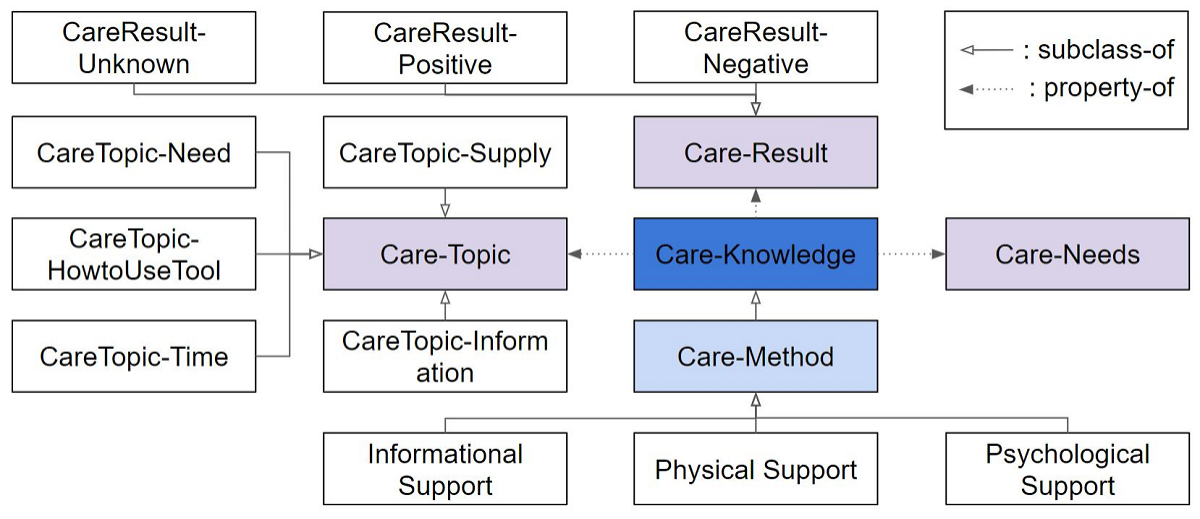
The structure of the “Care Knowledge” class in care ontology.

The detailed structure of the Care Knowledge class is shown in Figure 4. The Care Knowledge class has three different subclasses: Informational Support, Physical Support, and Psychological Support. The Care Knowledge class has the properties of Care-Topic, Care-Needs, and Care-Result, which are used to describe the details of the care method and the history of the care results provided by the caregivers.

### Care Knowledge Management System 

**Figure 5:**
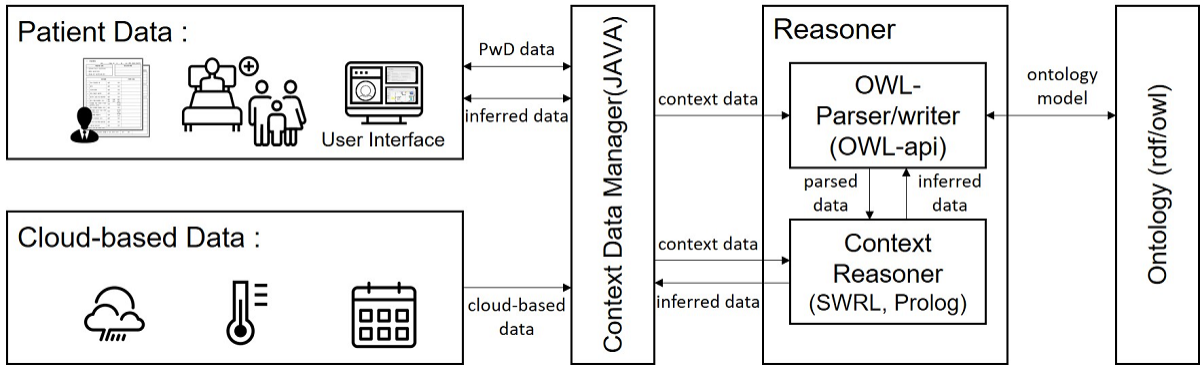
Overall architecture of the care knowledge management system. OWL: Web Ontology Language; PwD: persons with dementia; SWRL: Semantic Web Rule Language.

We developed a care knowledge management system based on the knowledge model of caring for persons with dementia so that caregivers can access appropriate care guides and manage the care information through a GUI on personal computers. The overall architecture of the proposed system is shown in Figure 5. The knowledge management system can utilize cloud-based data such as weather and calendar information from Google. The context data manager handles the incoming queries from the user application and passes on the care knowledge inferred by the reasoner module. The reasoner is equipped with two different reasoning rules as described in the Methods section. The SWRL is defined for care ontology and is used for inferring general care methods. The Prolog reasoner infers individualized care methods for a particular patient based on the knowledge described in the assessment class that contains the physical and psychological conditions of the individual with dementia.

**Figure 6:**
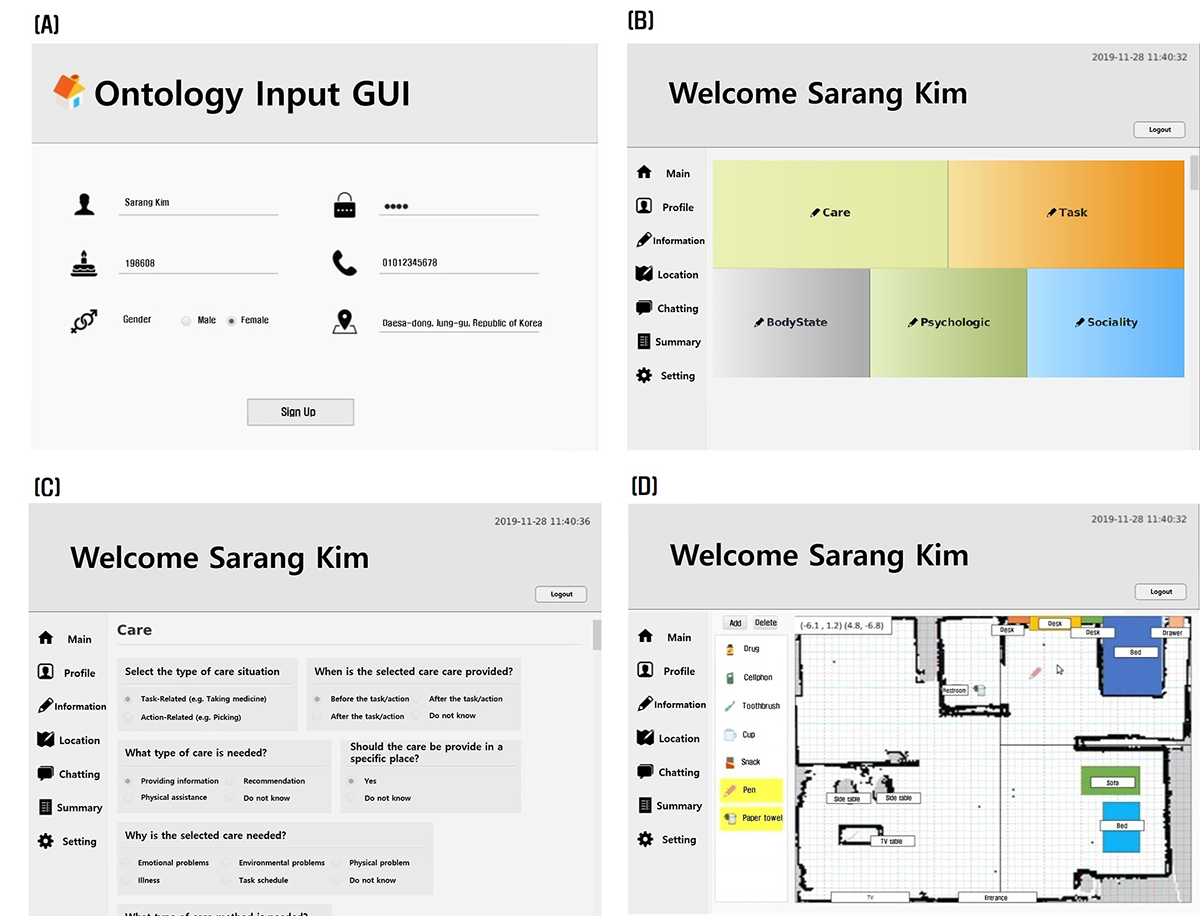
The graphical user interface design for care knowledge management: (A) an example of writing personal information on registration page, (B) selecting one of five different categories of assessment for persons with dementia, (C) updating assessment instances for care method, and (D) checking and updating the location of objects.

The care knowledge management system can be accessed via a GUI where the caregivers can manage or review instances of the ontology (Figure 6). For example, instances of Person class and Environment class can be accessed through the Profile and Location sections, respectively. To add new instances or to update the existing instances of assessment, the user needs to answer the list of questions corresponding to the selected topic (Care Method, Task, Body State, Psychological State, and Sociality). All the questions were created based on the actual assessment document used at the care center in Korea. Figures 6B and 6C show an example of questions on Care Method and instances of objects contained in the ontology.

### Case Study: Instances of Persons With Dementia

Table 1 presents a summary of the actual information for people with dementia written in a care manual obtained from a care center in Korea (we modified personal information such as name, address, and phone number). Ms Park has been diagnosed with mild dementia and is currently taking medication for chronic high blood pressure. She shows memory impairment symptoms such as not remembering the location of her drugs and the sequence of daily activities (eg, using the toilet). She can eat meals by herself but cannot control her appetite. The care manual also contains care guidelines for dealing with the individual’s symptoms, such as helping her to remember an object’s location and to flush the toilet bowl. Mr Kim has been diagnosed with mild dementia and is currently taking medication for diabetes. He has particular problems with memory impairment and regulation of his emotions.

**Table 1:** Assessment information on people with dementia obtained from the care center in Korea. Personal information such as name, gender, and address are modified.

Item	Example 1	Example 2
Name	Sooji Park	Inja Kim
Gender	Female	Male
Age (years)	70	80
Address	Jongno-gu, Seoul	Sungin-dong, Seoul
Contact	010-1234-0000	010-9876-0000
Dementia condition	Mild dementia	Mild dementia
Areas that need care	When she takes her high blood pressure medication, caregiver must remind her of the location of the medication. She eats well but cannot control her appetite. Caregiver should remind her to flush the toilet.	When he takes medicine, caregiver must remind him where the drug is and whether or not he has taken the medicine. He takes his diabetes drug three times a day after meals. When he moves to the bedroom, bathroom, or kitchen, he needs the caregiver’s physical support. He cannot remember the number of meals taken.
Mental illness and symptoms	She has emotional anxiety. She has hallucinations and delusions.	He is emotionally unstable. He can suddenly become angry and sometimes throws objects violently.
Physical illness	She has high blood pressure.	He has diabetes and knee pain.
Activity	She goes to church every Sunday. She likes to clean her room.	He goes to church every weekend.
Others	She uses diapers at night. She uses dentures.	He uses dentures. A picture of his family helps to cheer him up.

**Figure 7:**
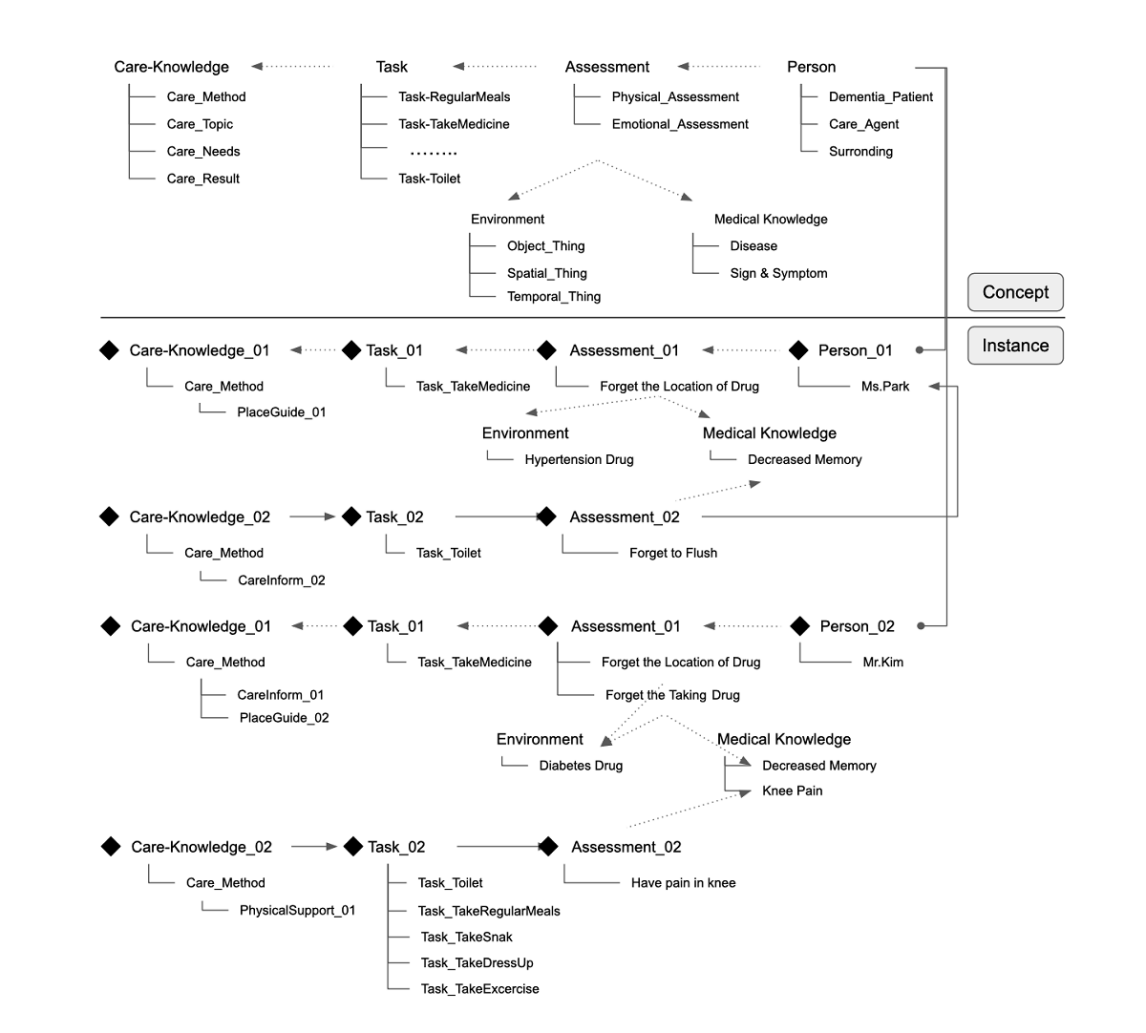
The structure of instances of a person with dementia together with upper-level concepts of care knowledge.

We used the knowledge management system to add the information for Ms Park, and Figure 7 illustrates the instances of her data together with the structure of the upper concepts in the ontology. The individual’s name, gender, age, address, and contact information are represented as instances of the Person class. Her mild dementia information, mental illness, and physical illness are represented in the Medical Knowledge class. Her task-related abilities, activities, and other precautions are expressed in the Assessment class. The care knowledge for informing on the location of the patient’s hypertension drug is expressed as an instance, Assessment_01 with Care-Knowledge_01. The care knowledge of the patient’s defecation activity is represented in Assessment_02 with Care-Knowledge_02. Care-Knowledge_02 thus expresses notification about using an object as a care method related to the defecation activity of the person with dementia.

### Evaluation of Care Knowledge Management System

**Figure 8:**
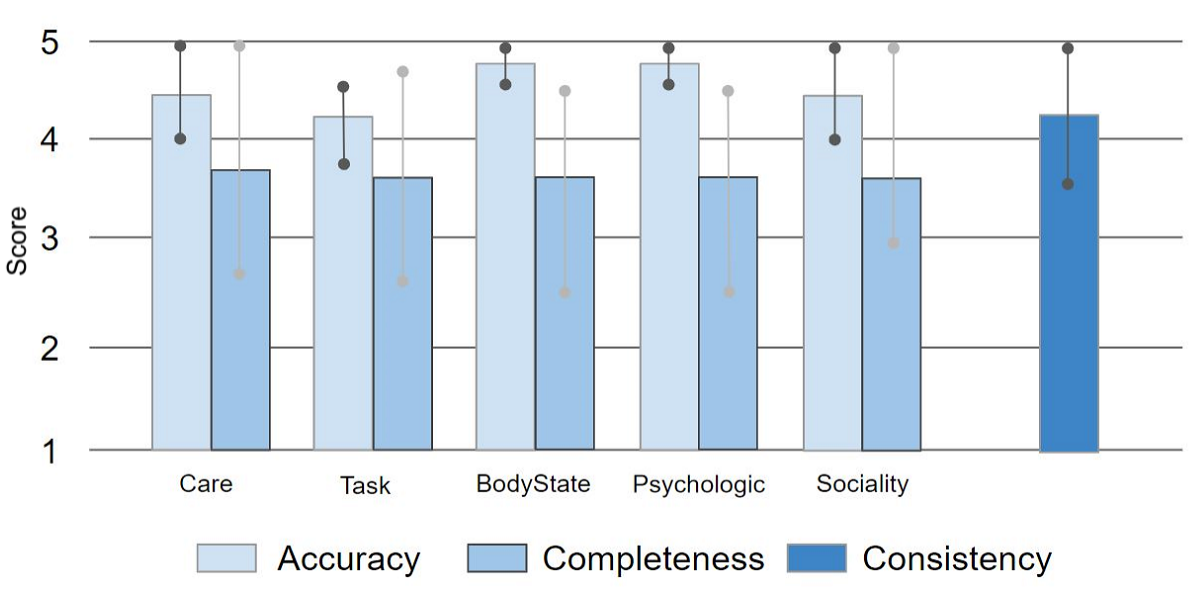
Evaluation of the care knowledge assessed by care experts in Korea.

We evaluated the care ontology with the OOPS! tool. We scored well on consistency, completeness, and conciseness, except for several minor issues such as a problem in determining the domain range and the property range for subclasses. In addition, we had our proposed system evaluated by 4 experts who had all been working in dementia care facilities for more than 10 years (the detailed procedure is described in the Methods section). The results of the evaluations received from experts are shown in Figure 8. To assess the accuracy of the ontology knowledge, we asked experts how accurately the proposed knowledge model described the actual knowledge used in a real care situation. In addition, in order to assess the completeness of the knowledge, we asked the experts whether the proposed system contained enough knowledge on caring for persons with dementia. In other words, the completeness score represents whether or not the knowledge model covers the necessary knowledge for caring for persons with dementia. Finally, regarding the consistency of the knowledge, the experts were asked whether or not the terms in the system developed were consistent with the terms used in the actual welfare centers. As a result, the caregivers evaluated that the current system contains accurate care knowledge (accuracy score: mean 4.55, SD 0.46) and covers most of the required care knowledge (completeness score: mean 3.80, SD 1.12). Regarding the consistency of the assessment information, the results show that the current ontology contains a comparable level of assessment information to the assessment tools (consistency score: mean 4.25, SD 0.83).

## Discussion

### Principal Findings

In this study, we built an ontological knowledge model for dementia care and developed a care knowledge management system that can provide care guidelines tailored to various behavioral and psychological symptoms of dementia. The care knowledge management system includes specific activity types of the people with dementia with different dementia symptoms and care methods that are based on their dementia assessment. With the system, caregivers can easily get basic information on the different dementia patients that they care for and can be assisted with a care guide for the different tasks of the individuals.

It is well known that the symptoms of dementia are an important source of the burden and stress carried by caregivers. There is also evidence that exposure to chronic stress in the caregivers of those with dementia is associated with decreased physical health, mental illness, and poor quality of life in the caregiver [31-33]. Due to the high stresses of the care, caregivers often neglect or even abuse the person with dementia [4]. Numerous interventions have been proposed to change caregivers’ behavior by increasing their social support and thereby improving their psychological state. There are home-based interventions, technology-driven interventions (ie, interventions that use computers or web-based applications), and interventions that are delivered via telephone. In a study by Godwin et al, 271 articles were reviewed for the efficacy of technology-driven interventions for the caregivers of persons with dementia [13]. They found that some of the studies showed reduced depression and anxiety among caregivers in the intervention group. However, the authors cautioned that no intervention method could completely reduce the burden on the caregiver because of the lack of technologies that monitored the patient's condition while supporting the caregiver.

Recent studies have attempted to provide better care service to persons with dementia by diagnosing their medical condition or by increasing their independence in daily activities. The DMSS-R supports the interventions performed by individual health professionals in daily practice with dementia patients [12]. DMSS-R is based on the Clinical Practice Guidelines for dementia diagnosis and provides diagnostic results, focusing on the physician's perspective on the patient. However, in addition to a clinical diagnosis of the dementia, caregivers need more information regarding the tasks of persons with dementia and their ability to perform these tasks. Other technologies, including a visual map and a memory notebook, help persons with dementia by presenting images relevant to their daily lives and schedules in context [14]. The visual map is a system that informs the individual of action guidelines through images and sound. It shows the necessary actions step by step through images that can help the person with memory impairment. However, there is a limit to the ability of persons with memory and behavioral impairments to live by themselves using only these systems without a caregiver's help. For example, an individual with dementia and physical disability could not be completely independent without the physical support of a caregiver, and this may inevitably require a care guide when making decisions.

We therefore focused on a situation where the caregivers could be relieved of remembering all the symptoms of the dementia patients they care for and of deciding on appropriate care methods for each one. To build a care knowledge system, we used the AAIS ontology and the K4Care ontology to obtain information on the disease types for dementia [8,10]. The AAIS ontology contains concepts of BPSDs that deal with the symptoms of impairment in perceptions, emotions, and behavior, while the K4Care ontology deals with the disease and the symptoms observed in the health care records of mild dementia patients. We included information about patients’ physical diseases and social situations in addition to knowledge from the AAIS and K4Care ontologies, which included intervention information. We acquired object and spatiotemporal information from the KnowRob ontology to express knowledge regarding objects, times, and places relating to the person with dementia [11]. Additionally, the care ontology included individual care methods obtained from care-related books and the actual care guidelines used in dementia care facilities. Therefore, the proposed care ontology could represent not only the patient’s disease symptoms, individual condition, and environmental information but also care methods suited to the different care situations.

To give caregivers easy access to the care knowledge and allow them to update information on the person with dementia, we developed a GUI for the knowledge management system (Figure 6). The GUI was designed for communication not only of personal information but also of symptoms, diseases, and social relationships that need to be considered when determining a care method. The system is able to manage and provide care-related knowledge for an unlimited number of persons with dementia and a large volume of additional care information. Although the main users of the proposed system would be professional caregivers in dementia care facilities, we believe that such a system could be easily extended so that nonprofessionals, such as family members, could access care information without special knowledge and that this would be beneficial by reducing both the burden and cost of caring for persons with dementia.

### Limitations

Based on our interviews with professional caregivers, we realized that caregivers also pay considerable attention to emotional factors when deciding how to care for a person with dementia, and this explained the relatively low scores achieved by the current ontology for completeness (Figure 8). The proposed care ontology only considers the environmental factors, task performance, physical illness, mental illness, and social activities of the dementia patient. Due to the highly complex situations that can affect a person's emotional state, it is not easy to provide suitable care guidelines for the different emotional states of individuals with dementia. Future studies should investigate the range of psychological symptoms exhibited by persons with dementia with the relevant care that should be performed in care facilities so as to include the emotional dimension in future care knowledge. Various ontological models of human emotions could also be examined [34,35]. In addition, the developed care knowledge model targeted patients with mild dementia, and the experts who evaluated it cared for patients with mild dementia. Since the knowledge model deals only with knowledge related to mild dementia patients, it would be less helpful for the caregivers of patients with severe dementia.

The decision rules defined in the proposed knowledge management system are based on care methods described in interviews with care experts and in care manuals used in a care facility in Korea. The “Care Knowledge” and “Assessment” classes include terms and concepts that are described in these care manuals. For this reason, the care guidelines outlined in the proposed system would only be applicable to caregivers working in care facilities in Korea. Further studies are needed to extend the ontological model and the decision rules so that the proposed system would apply in places outside of Korea.

Finally, in this study, the accuracy and consistency of the care knowledge and the usability of the proposed knowledge system were evaluated by only 4 different care experts, although these experts each had an average of 10 years of dementia care experience. In addition, it remains to be tested whether the current system could reduce the care burden and help with care decision making in real care conditions. Currently, we are preparing a web-based application that can be accessed through mobile systems such as a tablet PC or smartphone for more convenient access to the care knowledge management system, and we plan to apply this updated system in real care situations.

### Conclusions

In this study, we proposed a care knowledge management system that can provide the caregiver with detailed care information for the person with dementia. The system includes an ontological model of dementia care, a context data manager, and a rule-based reasoner in SWRL and Prolog. In addition, the system is equipped with a GUI, and it can be used anywhere in the home or welfare center regardless of the number or location of the dementia patients. Since the proposed system can provide care guidelines suitable for dementia patients, it is anticipated that the proposed system could reduce the workload of caregivers, but it remains to be tested in a future study.

## Abbreviations

AAIS: ambient-assisted intervention system
BPSDs: behavioral and psychological symptoms of dementia
DMN: digital memory notebook
DMSS-R: Dementia Management and Support System
GUI: graphical user interface
OOPS!: OntOlogy Pitfall Scanner!
OWL: Web Ontology Language
SWRL: Semantic Web Rule Language
